# SUMO1 Modification of Tau in Progressive Supranuclear Palsy

**DOI:** 10.1007/s12035-022-02734-5

**Published:** 2022-05-14

**Authors:** Hironori Takamura, Yoshiaki Nakayama, Hidefumi Ito, Taiichi Katayama, Paul E. Fraser, Shinsuke Matsuzaki

**Affiliations:** 1 Tanz Centre for Research in Neurodegenerative Diseases, University of Toronto, Toronto, ON Canada; 2 Department of Child Development & Molecular Brain Science, Center for Child Mental Development, United Graduate School of Child Development, Osaka University, Osaka, Japan; 3 Department of Neurology, Wakayama Medical University, Wakayama, Japan; 4 Department of Medical Biophysics, University of Toronto, Toronto, ON Canada; 5 Department of Radiological Sciences, Faculty of Health Sciences, Morinomiya University of Medical Sciences, Osaka, Japan

**Keywords:** Tauopathy, Truncated tau, Small ubiquitin-like modifiers (SUMO), Neurodegeneration, Protein aggregation, Neurofibrillary tangles

## Abstract

Small ubiquitin-like modifiers (SUMO) have been implicated in several neurodegenerative diseases. SUMO1 conjugation has been shown to promote aggregation and regulate phosphorylation of the tau protein linked to Alzheimer’s disease and related tauopathies. The current study has demonstrated that SUMO1 co-localizes with intraneuronal tau inclusions in progressive supranuclear palsy (PSP). Immunoprecipitation of isolated and solubilized tau fibrils from PSP tissues revealed SUMO1 conjugation to a cleaved and N-terminally truncated tau. The effects of SUMOylation were examined using tau-SUMO fusion proteins which showed a higher propensity for tau oligomerization of PSP-truncated tau and accumulation on microtubules as compared to the full-length protein. This was found to be specific for SUMO1 as the corresponding SUMO2 fusion protein did not display a significantly altered cytoplasmic distribution or aggregation of tau. Blocking proteasome-mediated degradation promoted the aggregation of the tau fusion proteins with the greatest effect observed for truncated tau-SUMO1. The SUMO1 modification of the truncated tau in PSP may represent a detrimental event that promotes aggregation and impedes the ability of cells to remove the resulting protein deposits. This combination of tau truncation and SUMO1 modification may be a contributing factor in PSP pathogenesis.

**Supplementary Information:**

The online version contains supplementary material available at 10.1007/s12035-022-02734-5.

## Introduction

Small ubiquitin-like modifiers (SUMOs) exist as five isoforms. SUMOs function to regulate proteins in several ways including subcellular localization, nuclear transport, cell migration, transcriptional repression and cell-cycle progression [1–4]. Target proteins are SUMOylated at lysine residues [1, 5].

In the field of neuroscience, a lot of findings have reported the importance of SUMOs, especially SUMO1 and SUMO2/3, as described below and SUMO1 shares ~ 50% homology with SUMO2 and SUMO3 while SUMO2/3 are virtually identical [6]. SUMO is involved in a number of neuronal and synaptic pathways such as the regulation of myocyte enhancer factor 2 (MEF2) that modulates neurite outgrowth and synaptic plasticity [7]. A recently generated SUMO1 transgenic model has demonstrated a significant contribution of SUMOylation to the development and maintenance of dendritic spines [8]. The microtubule-associated protein tau is another neuronal protein modified by SUMO1 [9]. Tau is a natively unfolded phosphoprotein localized to axons and controls microtubule stability and axonal development [10]. Human tau is encoded by the MAPT gene on chromosome 17p21 and contains 16 exons to produce at six major isoforms through alternative splicing (for reviews see, [11]). Tau is preferentially SUMOylated by SUMO1 at the lysine residue 340 (K340) which is located in the fourth (4R) microtubule-binding repeat [9]. SUMOylation of tau is enhanced by treatment with the phosphatase inhibitor okadaic acid or colchicine, a microtubule depolymerizing drug, suggesting SUMO preferentially interacts with phosphorylated tau. Furthermore, conjugation of SUMO1 to the tangle-related tau protein induces hyperphosphorylation and inhibits tau degradation by the ubiquitin-proteasome pathway [12]. Proteomic analyses of transgenic mouse models have confirmed that tau is a major in vivo target for SUMO1 which may impact its neuronal and glial functions [8].

Tauopathies are characterized by the accumulation of abnormal hyperphosphorylated tau as neurofibrillary tangles (NFTs) [13]. Tauopathies encompass a broad range of disorders including Alzheimer’s Disease (AD), Pick’s Disease, Frontotemporal Dementia with Parkinsonism (FTDP-17), corticobasal degeneration (CBD), chronic traumatic encephalopathy (CTE) and progressive supranuclear palsy (PSP) [13–16]. PSP is a sporadic neurodegenerative disorder which presents clinically with vertical (supranuclear) gaze palsy, rigidity and postural instability [17]. PSP is associated with a variety of intracellular tau aggregates such as neurofibrillary tangles, neuropil threads and glial fibrillary tangles leading to extensive brain atrophy (for reviews see, [18, 19]). PSP is also associated with truncated tau where the N-terminus (~ 160 residues) have been proteolytically cleaved [20]. The functional relevance of these truncated tau species has been shown to increase microtubule stabilization, tau aggregation and contributions to AD pathology [21, 22]. SUMO1 is also associated with lysosome and suggested to have a role in lysosomal function which is associated with glial protein aggregation [23].

SUMOylation has been linked to several neurodegenerative diseases and may be directly involved in promoting the deposition and/or preventing the clearance of intracellular protein aggregates [24–26]. Several of the aggregation-prone proteins involved in neurodegenerative disorders including α-synuclein, tau and huntingtin are modified by SUMO [9, 27–30]. The consequences of SUMOylation for the formation of these inclusions are currently under debate as, for example, α-synuclein aggregation can either be prevented or promoted by SUMOylation under difference conditions [28, 29, 31]. The present study examines the role of SUMOylation on tau accumulation in PSP which demonstrated that pathological tau inclusions are immunoreactive for SUMO1. In addition, biochemical analyses of isolated tangles reveal that the N-terminally truncated PSP-related tau is covalently modified by SUMO1. In vitro examination of SUMO-tau fusion proteins indicates that SUMO1 specifically increases tau aggregation, particularly that of the truncated tau associated with PSP. These findings suggest that SUMOylation of tau might be a contributing factor to the aggregation and accumulation of neuronal and glial tau possibly by altering protein solubility and/or degradation.

## Materials and Methods

### Antibodies

Peptide antigens used for polyclonal antibody generation corresponded to SUMO1 C-terminus residues 73–97 (IADNHTPKELGMEEEDVIEVYQEQT) or SUMO2 N-terminal residues 3–24 (EEKPKEGVKTENDHINLKVAGQ) as previously described [8]. Peptides were covalently linked to keyhole limpet hemocyanin (KLH) and used to immunize rabbits. Polyclonal antibodies from collected and pooled antisera were affinity-purified as previously described [32]. Immunoblotting of lysates from cells expressing HA-tagged SUMO1, 2 or 3 confirmed the specificity of these polyclonal antibodies [8]. Polyclonal rabbit anti-human tau antibody (DAKO, Carpinteria, CA, USA, A0024), monoclonal mouse anti-SUMO1 antibody (Invitrogen, Eugene, OR, USA, 33–2400), monoclonal rabbit anti-SUMO1 (Abcam, Cambridge, UK, ab32058, clone Y299), monoclonal mouse anti-phospho-tau (Ser202, Thr205) AT8 antibody (Thermo Fischer, Waltham, MA, USA, MN1020), monoclonal mouse anti-4-repeat tau (Sigma-Aldrich, St. Louis, MO, USA, 05–804 RD4, clone 1E1/A6), monoclonal mouse anti-glial fibrillary acidic protein (GFAP) (Merck Millipore, MAB360, clone GA5), polyclonal rabbit anti-Grp7/BiP antibody (Enzo, Farmingdale, NY, USA, ADI-SPA-827), monoclonal mouse anti-β tubulin antibody (Sigma-Aldrich, T8328), monoclonal mouse anti-Vimentin antibody (Abcam, ab8978); and a monoclonal mouse anti-β-actin antibody (Sigma-Aldrich, A5316); were used in this study. Secondary antibodies were: Cy3-conjugated anti-rabbit IgG antibody (Jackson ImmunoResearch Laboratories, Inc., West Grove, PA, USA, 111–165-144); and FITC-conjugated anti-mouse IgG antibody (Jackson ImmunoResearch Laboratories, Inc., 115–095-146). For histological studies, the antibodies included SUMO1 (Abcam, ab32058, clone Y299, rabbit monoclonal); anti-4-repeat tau (Sigma-Aldrich, 05–804 RD4, clone 1E1/A6, mouse monoclonal) and anti-p tau (AT8, Thermo Fischer, MN1020). Secondary antibodies included Alexa Fluor 488 (Donkey anti-mouse, Thermo Fisher, A21202,) and Alexa Fluor 647 (Donkey anti-rabbit, Thermo Fisher, A-31573).

### Immunohistochemistry

Brain tissues from individuals with clinical diagnosis of PSP and healthy control were kindly provided by Dr. Gabor Kovacs (University of Toronto), Dr. Dennis Dickson (Mayo clinic) and Dr. Hidefumi Ito (Wakayama medical university). We used tissues from eight PSP, three control and total 39 slides for data analysis. Tissues were fixed in 10% buffered formalin and standard blocks were taken from basal ganglia, embedded in paraffin and sectioned (15 μm). Tissue sections were first deparaffinized with xylene and washed in serial dilutions of ethanol and immersed in pH 6.0 antigen retrieval solution (Nichirei Biosciences, Tokyo, JAPAN) at 120 °C for 20 min in a pressure cooker. Then, the sections for 4-repeat tau staining were incubated in 90% formic acid for 5 min. For double-immunofluorescent staining, after 1 h incubation in blocking solution composed of 3% bovine serum albumin (BSA) in PBS, sections were transferred into PBS containing the primary antibodies against SUMO1 (Abcam; 1:250) and p tau (1:500), SUMO1 (Abcam; 1:250) and 4-repeat tau (Sigma-Aldrich; 1:1000), SUMO2 (1:1500) and 4-repeat tau (1:1000), SUMO1 (Abcam; 1:250) and GFAP (Merck Millipore; 1:800) and kept at 4 °C overnight. After washing with PBS, the sections were incubated in the secondary antibody solution containing 3% BSA, Alexa Fluor 488 (1:150) and Alexa Fluor 647 (1:150) conjugated secondary antibodies in PBS at 4 °C for 3 h. The sections were also incubated in 70% ethanol with 0.3% Sudan Black B for 10 min, and cover slips applied with VECTASHIELD Hard Set (Vector Laboratories, Burlingame, CA, USA). For immunohistochemistry, anti-SUMO1 (1:250) or anti-SUMO2 (1:1500) were generated as described previously [8] or anti-p tau (AT8, MN102; 1:500) was added to the primary antibody solution. The sections were incubated in N-Histofine Simple Stain MAX PO (peroxidase) reagent (Nichirei) for 30 min and visualized by DAB (Vector DAB, Vector laboratories). All sections were counterstained with hematoxylin, dehydrated in a graded series of ethyl alcohol, cleared in xylene and cover slips applied, as above.

### Immunoblot Analyses

For analysis of SUMO1 levels in disease cases, human control, AD and PSP brain samples were solubilized in lysis buffer (50 mM HEPES-KOH pH 8.0, 100 mM KCl, 2 mM EDTA, 0.5% NP-40, 1% SDS, 10% glycerol and protease inhibitors) and equal amounts of total protein (40 µg) from each sample were examined by immunoblotting for proteins of interest. Immunoblotting was performed using Tris-glycine gels 4–20% (Invitrogen) and transferred onto nitrocellulose. For cell culture study, we used at least three samples for each condition. Cells were washed twice in phosphate-buffered saline (PBS), harvested by scraping and lysed with RIPA buffer (50 mM Tris–HCl, 1 mM EDTA and 150 mM NaCl, 1% Triton X-100, 0.5% deoxycholate, 0.1% SDS) containing protease inhibitor cocktail (Roche Diagnostics, Mannheim, Germany). Equal amounts of protein were subjected to SDS-PAGE and transferred to nitrocellulose membranes. The membranes were blocked with 10% (w/v) skim milk in TBS and then incubated with TBS containing 1% skim milk, 0.2% tween-20 and primary antibody against tau (1:100,000), SUMO1 (rabbit polyclonal, 1:1000), SUMO2 (1:1000) or β-actin (1:50,000), followed by incubation with an HRP-conjugated secondary antibody. Signals were detected by enhanced chemiluminescence (ECL).

### Isolation of Neurofibrillary Tangles

Fibrous tau inclusions were isolated as previously described [33]. Briefly, brain tissue lysates were prepared in 100 mM MES; 0.75 M NaCl; 1 mM EGTA; 0.5 mM MgSO4; pH 6.5 with protease inhibitors (1:10 w/v ratio) using a Dounce homogenizer. Homogenates are incubated at 4 °C for 20 min to depolymerize any residual microtubules and centrifuged at 11,000 g for 20 min at 4 °C. Supernatants were spun at 100,000 g for 1 h at 4 °C and pellets were suspended in 10 mM Tris buffer (pH 7.4) containing 10% sucrose, 0.85 M NaCl and 1 mM EGTA. Samples were centrifuged at 15,000 g for 20 min at 4 °C and supernatants were suspended in Sarkosyl (1% final concentration). Purified tau tangles were isolated by centrifugation at 100,000 g for 30 min. Negative-stain transmission electron microscopy of the isolated tau aggregates was performed as previously described [34].

### Immunoprecipitation

Isolated tangle preparations were solubilized in buffer (10 mM Tris, pH 7.4; 0.85 M NaCl; 1 mM EGTA) containing 2% SDS and the solution was clarified by brief centrifugation (15 min at 15,000 g). Samples were diluted to 0.1% and lysates were first subjected to preclearing with protein G-Sepharose 4 fast flow (GE Healthcare, Uppsala, Sweden). SUMO1 polyclonal antibody conjugated to protein G-Sepharose beads were added to the lysates and incubated overnight at 4 °C. Immunoprecipitated proteins were eluted with in Laemmli buffer and resolved by SDS-PAGE (4–12% Bis–Tris gels, BioRad, Hercules, CA, USA) and probed with tau antibody (DAKO, A0024; 1:10,000).

### Tau-SUMO Fusion Proteins

Full-length 4-repeat (4R) tau plasmids were as previously described [9]. The N-terminally truncated tau spanning residues 160–441 as well as the truncated tau-SUMO1 and tau-SUMO2 fusion proteins were generated using a PCR fragment from the full-length proteins. Expression was facilitated by the insertion of an ATG start-codon on the N-terminal side of Arg-161 and inserted into a pcDNA3 vector.

### Cell Culture and Transfection

HeLa cells and HEK cells were cultured in DMEM (Invitrogen) containing 10% fetal bovine serum (FBS) at 37 °C and 5% CO_2_. Cells were transfected with the required tau construct and Lipofectamine 2000 (Invitrogen) according to the manufacturer’s protocols.

### Immunocytochemistry

HeLa cells were fixed with 4% paraformaldehyde in PBS for 30 min at room temperature. After 1 h incubation in blocking solution comprising 5% bovine serum albumin (BSA), 0.3% Triton X-100 in PBS, the cells were incubated in the same solution containing primary antibodies against tau (1:10,000) and Bip (1:200), β-tubulin (1:400) or vimentin (1:400) at 4 °C for 24 h. The cells were incubated in the secondary antibody solution containing 5% BSA and Cy3-conjugated anti-rabbit IgG antibody (1:400) and FITC-conjugated anti-mouse IgG antibody (1:400) at room temperature (RT) for 1 h. The coverslips were mounted onto slides using ProLong Diamond Antifade Mountant with DAPI (Invitrogen, P36962). Fluorescence images were acquired using Keyence BZ-9000 and Nikon Eclipse Ti. For quantification of tau localization, 100 cells/slide and 5 independent slides for each condition were assessed (total 500 cells/condition).

### Epoxomicin Treatment and Cellular Fractionation

HEK293 and HeLa cells at 24 h post-transfection were treated with 0.1 µM or 1 µM Epoxomicin (Sigma E3652). After incubation for 24 h, the cells were washed with PBS and harvested. Cells were lysed in TBS containing 1% Triton X-100, 1% sodium deoxycholate, 50 mM NEM and protease inhibitor cocktail then kept on ice for 30 min followed by centrifugation at 15000 g for 20 min at 4 °C. Supernatants were collected as soluble fraction and pelleted insoluble fraction was lysed in RIPA buffer as insoluble fraction prior to immunoblot analysis. For cytosolic/nuclear fractionation, harvested cells were resuspended in TNE buffer (50 mM Tris–HCl, pH 7.8, 5 mM EDTA, 1% NP-40 and 150 mM NaCl) containing a protease inhibitor cocktail (Roche Diagnostics). Cells were centrifuged at 1000 × g for 10 min at 4 °C and the supernatant was collected as cytosolic fractions. The pellets were resuspended in RIPA buffer, sonicated and centrifuged at 10,000 × g for 10 min to clear the nuclear fraction.

### Cell Viability Assay

HEK293 cells were seeded at a density of 5.5 × 10^3^ cells in each well of 96-well plates and transfected as mentioned above after 24 h, and then, cells were treated with DMSO or Epoxomicin for 24 h. Cell viability was analyzed by CellTiter-GLO 2.0 (Promega, Madison, WI, USA) according to the manufacturers’ protocol. Signals were measured using the Centro XS^3^ LB960 (Berthold Technologies, Bad Wildbad, Germany). For measurement of cell viability, 6 wells/96-well plate and 10 independent plates for each conditioned cells were assessed.

### Statistical Analysis

For statistical analyses, Student’s *t* test by MS Excel has been used when comparing two groups.

## Results

### SUMOylation of PSP Tau Inclusions

**Fig. 1:**
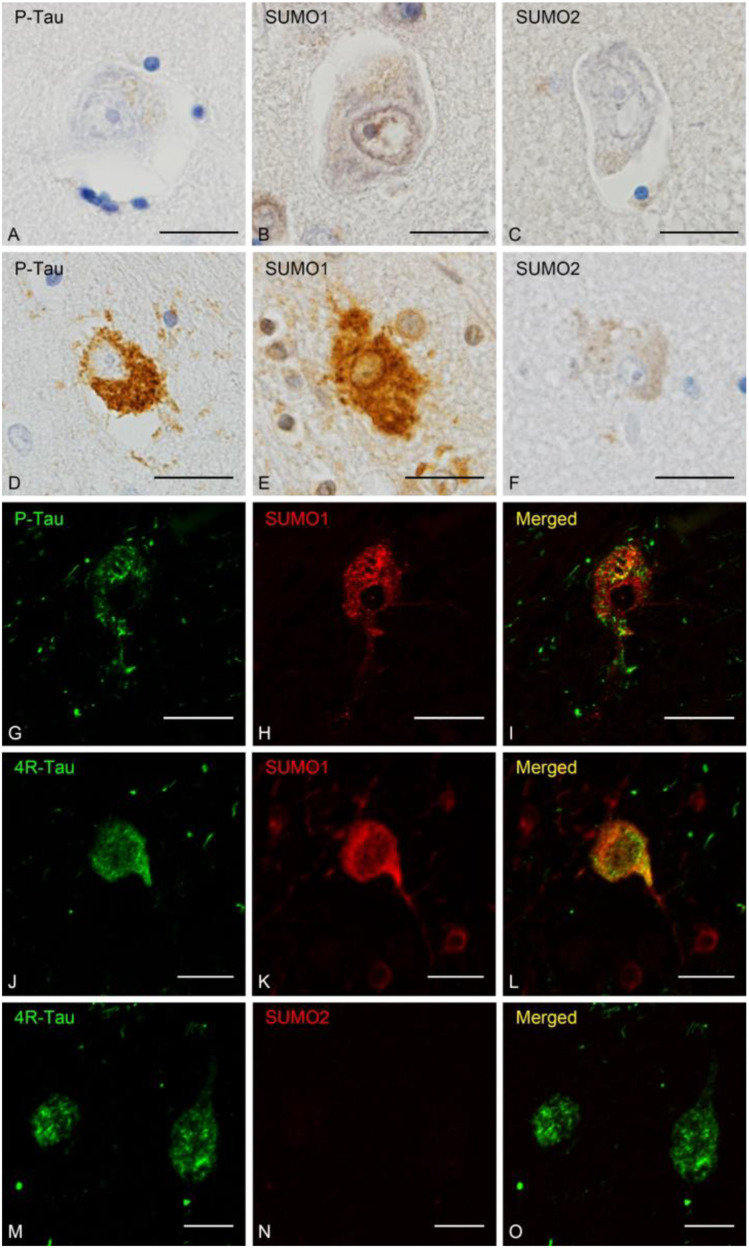
Neuronal Tau Pathology and SUMO. Control and PSP tissues were stained for tau and examined for its colocalization with SUMO1 and SUMO2. Immunohistochemistry for **(A)** phosphorylated tau (P-Tau), **(B**) SUMO1 and **(C)** SUMO2 in control tissues. Immunohistochemistry for **(D)** P-Tau, **(E**) SUMO1 and **(F)** SUMO2 in PSP tissues. Double labeling immunofluorescence in PSP tissues for **(G)** P-Tau, **(H)** SUMO1 and **(I)** merged. Immunofluorescence staining in PSP tissues for **(J)** 4-repeat tau (4R-Tau), **(K)** SUMO1 and **(L)** overlapping immunoreactivity. Staining in PSP tissues for **(M)** 4R-Tau, **(N)** SUMO2 and **(O)** merged 4R-Tau and SUMO2. Scale bars are 20 μm

The relationship between tau and SUMOylation was examined in control and PSP cases by immunohistochemistry and immunofluorescence (Table 1). Immunohistochemistry for phosphorylated tau (P-Tau) (Fig. 1A), SUMO1 (Fig. 1B) and SUMO2 (Fig. 1C) in the basal ganglia did not exhibit any characteristic staining except faint glial cytoplasmic immunoreactivity in control brain, on the other hand, PSP brain showed perinuclear accumulations of P-Tau in neurons consistent with globose type neurofibrillary tangles commonly observed in PSP (Fig. 1D) [35]. Probing for SUMO1 in the adjacent brain region indicated similar strong immunoreactivity within the neuronal cytoplasm that resembled aggregated tau depositions (Fig. 1E). In contrast, only modest and diffuse reactivity was observed for SUMO2 (Fig. 1F). Comparable immunofluorescence for P-Tau and SUMO1 revealed punctate reactivity for both proteins but only a small degree of overlap between the two species (Fig. 1G-I). A more extensive overlap was observed for the 4-repeat tau (4R-Tau) and SUMO1, particularly within the perinuclear and the axonal hillock (Fig. 1J-L). Consistent with the immunohistochemistry results, SUMO2 immunoreactivity was much lower than that of SUMO1 in these cases (Fig. 1M-O). This may indicate that SUMO1 is preferentially associated with pre-tangle or protofibril forms of tau in PSP.

**Table 1:** Clinical details on the unaffected controls, progressive supranuclear palsy (PSP), Alzheimer disease (AD) tissues that were investigated to determine the expression levels of SUMOl conjugates in this study

Case ID	Pathological Diagnosis	Age	Sex
NA09-051	PSP	62	M
NA10-226	PSP	80	M
NA10-384	PSP	64	F
NA10-431	PSP	75	F
NA13-269	PSP	69	F
NA13-275	PSP	66	M
72–07-12	PSP	77	M
139–13-8	PSP	76	F
1523	PSP	68	M
1669	PSP	66	F
8812–052	Control	80	F
8813–028	Control	82	F
8814–010	Control	71	F
1110	Control	74	F
1361	Control	70	F
1094	AD	74	F
1183	AD	82	F

**Fig. 2:**
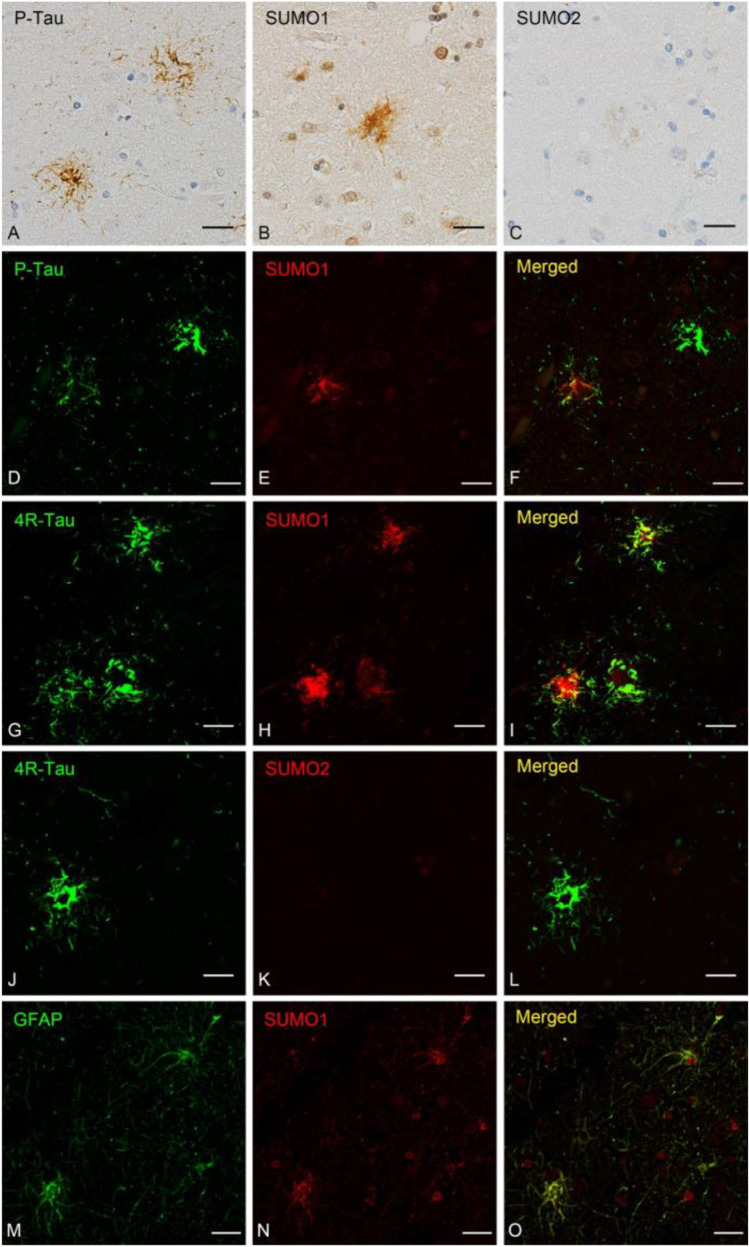
Astrocytic Tau Pathology and SUMO. PSP tissues were stained for tau and examined for its colocalization with SUMO1 and SUMO2. Immunohistochemistry for **(A)** phosphorylated tau (P-Tau), **(B)** SUMO1 and **(C)** SUMO2. Double labeling immunofluorescence staining for **(D)** P-Tau, **(E)** SUMO1 and **(F)** overlapping staining. **(G)** 4-repeat tau (4R-Tau), **(H)** SUMO1 and **(I)** overlapping staining. Labeling for **(J)** 4R-Tau, **(K)** SUMO2 and **(L)** merged staining. (**M**) Astrocytic Marker GFAP was also stained with (**N**) SUMO1 and (**O**) merged photo was shown. Scale bars are 20 μm

Examination of the glial associations of tau and SUMO showed the PSP-related tufted astrocytes that were positive for phospho-tau (Fig. 2A)[36]. Similar astrocytic immunoreactivity was also observed for SUMO1 in PSP suggesting a comparable accumulation of SUMO1 modified proteins (Fig. 2B). However, only low-intensity staining was observed for SUMO2 (Fig. 2C). Compared to neurons, both the P-Tau (Fig. 2D-F) and the 4R-Tau (Fig. 2G-I) exhibited less overlap with SUMO1 which may be due to accumulation of SUMO1 conjugated proteins other than tau in PSP astrocytes. Immunofluorescence probing for SUMO2 indicated decreased intensity in astrocytes and no co-localization with P-Tau was observed in these cases (Fig. 2J-L). We also confirmed that staining of SUMO1 was overlapped with GFAP, an astrocytic marker (Fig. 2M-O). Cumulatively, these results are consistent with a strong association of pathological tau and SUMO1 in affected neurons in PSP but SUMO1 weak modification of tau is seen in astrocytic pathology and glial SUMO1 may be linked to other aggregated proteins in non-neuronal cells.

### SUMO1-Conjugation to Tau in Isolated PSP Tangles

**Fig. 3:**
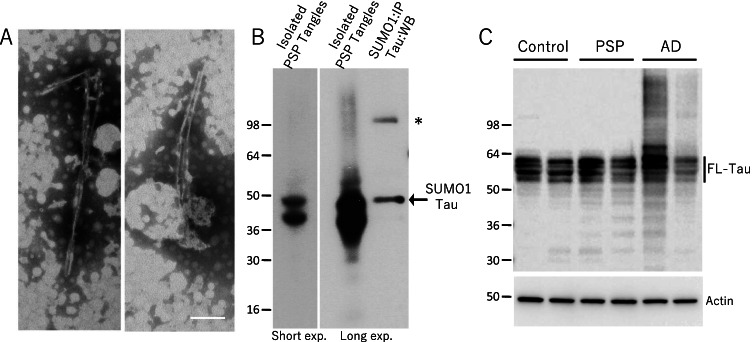
SUMOylation of Tau Inclusions**.** The filamentous inclusion in PSP tissues were isolated and examined by **(A)** negative-stain transmission electron microscopy which demonstrated the typical fibrils associated with tau inclusions. **(B)** Western blotting of the solubilized aggregates indicated a tau-positive doublet at 40–50 kD consistent with truncated forms of tau that are found in PSP. Immunoprecipitation of SUMO1-conjugated proteins in these isolated aggregates followed by western blotting for tau confirmed that Tau-SUMO1 conjugate band. Asterisk shows the appearance of taupositive higher molecular weight bands potentially as a result of protein misfolding and aggregation. **(C)** Tau and actin immunoblotting of total brain lysates from control, AD and PSP tissues (IP input) indicating the presence of the tau isoforms at a higher molecular weight as compared to SUMOylated tau consistent with a truncated tau in PSP being the major tangle species. Scale bar in (A) is 100 nm

The co-localization of SUMO1 and tau staining in PSP could conceivably be due to a direct modification of tau by SUMO1. To examine this, tau inclusions were isolated from PSP tissues (Table 1) using a series of differential solubility extractions and fractionation as previously described [33]. Negative-stain electron microscopy revealed that the isolates had fibrillar morphology (Fig. 3A). The filamentous or protofibril structures were solubilized and examined by immunoprecipitation and immunoblotting.

Probing for tau indicated two major bands at approximately 50 and 40 kD that represented the major species found in the isolated tau fibrils from the PSP tissue (Fig. 3B). The purified and solubilized tau filaments were subjected to immunoprecipitation using a SUMO1 polyclonal antibody followed by immunoblotting for tau. The 50 kD band was specifically precipitated by the anti-SUMO1 which is consistent with a covalent SUMOylation of the PSP tau-related inclusions (Fig. 3B). Interestingly, a higher molecular band at about 100 kD was detected and is potentially as a result of protein misfolding and aggregation (asterisk) (Fig. 3B). The PSP-related tau extracted from the fibrils exhibited a lower molecular weight than the full-length protein tau isoforms seen in unaffected controls as well as total lysates from PSP and AD tissues (Table 1, Fig. 3C). It is probable that the SUMOylated tau inclusions are proteolytically truncated which has been previously reported for PSP [37, 38]. Characterization of PSP tau indicated major species that is proteolytically cleaved to remove ~ 160 residues from the N-terminal which has been shown to be a prominent feature of PSP [37]. This combination of SUMO1 modification and the truncation of tau may result in a more aggregation-prone protein and also potentially interfere with its normal clearance by the ubiquitin-proteasome and lysosomal pathways. This could be one reason why SUMO1 may potentially have a greater impact on PSP than other tau-associated neurodegeneration disorders.

### Tau-SUMO Fusion Proteins as Models of PSP SUMOylation

**Fig. 4:**
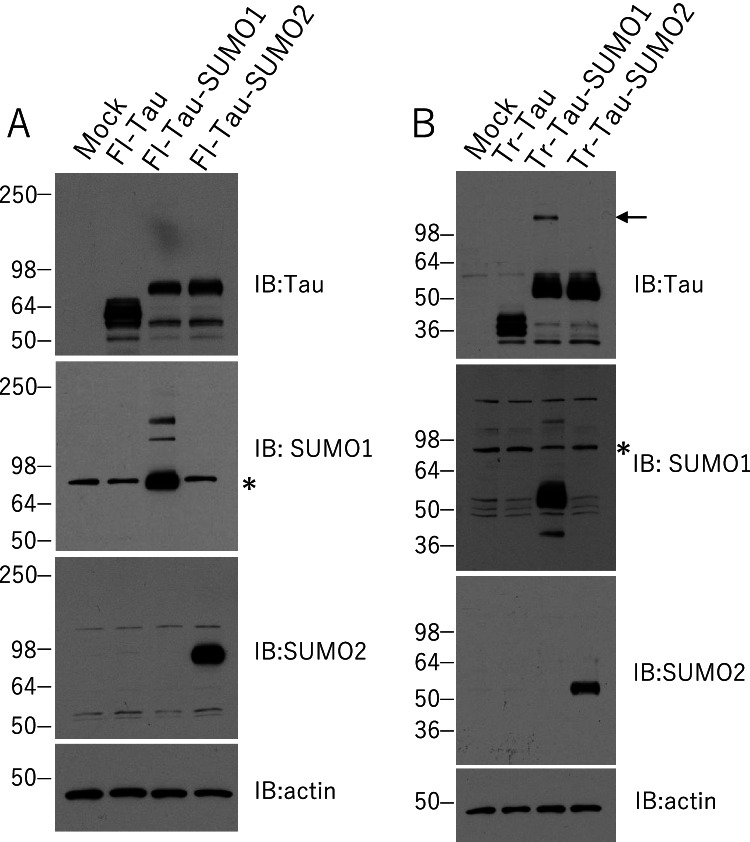
Tau-SUMO Fusion Proteins.** (A)** Full-length 4-repeat (4R) tau (Fl-tau) with SUMO1 or SUMO2 fused to its C-terminus probed for tau, SUMO1 and SUMO2 expressed in HEK293 cells. **(B)** Truncated tau (Tr-Tau) residues 160–441 similar to that associated with PSP with SUMO1 or SUMO2 fused to its C-terminus probed for tau, SUMO1 and SUMO2. The Tr-tau-SUMO1 expression resulted in the appearance of tau-positive higher molecular weight bands (*arrow*) potentially as a result of protein misfolding and aggregation. Asterisks shows highly SUMOylated RanGAP conjugates(~ 95kD) and the bands are overlapped with Tau-SUMO1 coincidentally

To examine the effects of SUMOylation on truncated tau in more detail, a series of fusion proteins were generated based on full-length (Fl-Tau) 4-repeat (4R) and the truncated PSP-associated tau (Tr-Tau) fragment lacking 160 amino acids from the N-terminal domain (truncated tau construct contains residues 161–441). The SUMO conjugation site is located near the C-terminus of tau (K340) and SUMO1 or SUMO2 were fused to C-terminal of the different tau species to mimic the physiologically SUMOylated proteins. Comparable SUMO fusion proteins have been employed previous to investigate the aggregation and prion-like activity of the cytoplasmic polyadenylation element-binding protein 3 (CPEB3) [39]. Tau-SUMO fusion proteins were expressed in HEK293 cells and immunoblotting indicated the expected bands for the Fl-Tau as well as the Tau-SUMO1 and Tau-SUMO2 fusion proteins (Fig. 4A). Probing for SUMO1 or SUMO2 confirmed the presence of the fusion proteins and an increase in Fl-Tau-SUMO1 higher molecular weight bands was also observed (Fig. 4A). These SUMO1-positive bands were not tau-positive, however, longer exposure of the immunoblots probed for tau did reveal a higher molecular weight band for the Fl-Tau-SUMO1 fusion protein which suggests that some aggregated species may be formed (data not shown). No higher molecular weight tau-immunoreactive species were observed for the unmodified Fl-Tau or Fl-Tau-SUMO2 fusion suggesting the addition of SUMO2 did not induce oligomerization of the fusion protein. In addition, a coincident band was observed in the SUMO1 blot likely corresponding to the RanGAP conjugate (~ 95kD) which is known to be highly SUMOylated [40].

The truncated tau (Tr-Tau) linked to PSP that was modified by SUMO1 (see, Fig. 3B) was also examined. The Tr-Tau with SUMO1 or SUMO2 fused to its C-terminus was expressed in HEK293 cells with comparisons made to Tr-Tau only. Probing for tau confirmed the expected fragment at ~ 36kD similar that found in the PSP brain lysates (Fig. 4B). Immunoblotting for SUMO1 or SUMO2 confirmed similar levels of the Tr-Tau-SUMO fusion proteins to cells expressing the unmodified truncated tau. The Tr-Tau-SUMO1 fusion protein exhibited the expect molecular weight (~ 52kD) as well as a high molecular weight band that could potentially arise from oligomers (Fig. 4B, arrow). In contrast, only a single tau-positive band was observed for the truncated tau-SUMO2 (Tr-Tau-SUMO2) fusion protein. These findings suggest that SUMO1 modification coupled with the N-terminal truncation of tau in PSP may lead to instability of tau folding and promote oligomerization.

**Fig. 5:**
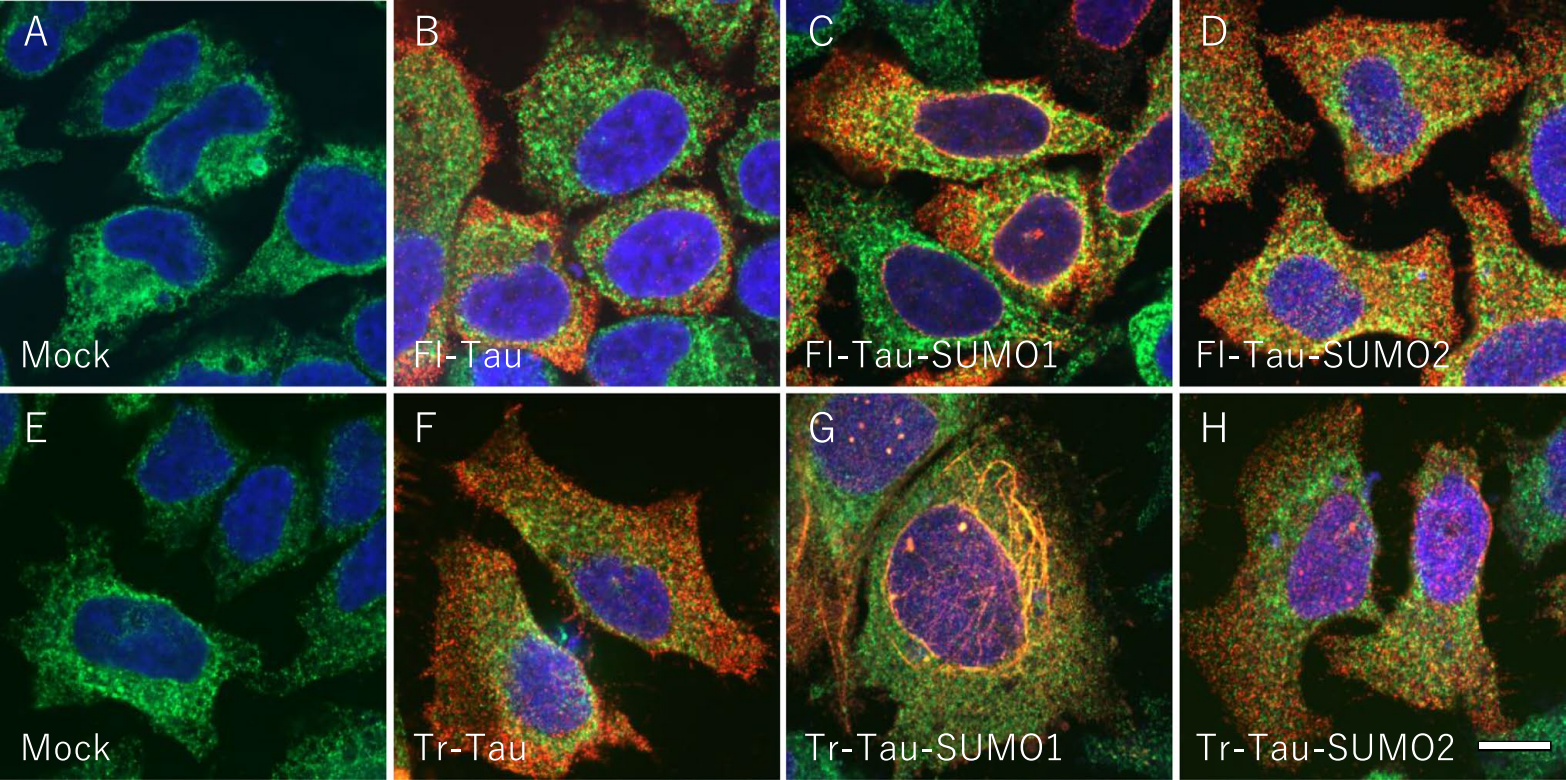
SUMO1 and Tau Aggregation. Subcellular localization of full-length and truncated tau and tau-SUMO fusion proteins were examined by immunofluorescence in HeLa cells. Cells were stained for the ER-marker BiP (green), tau (red) and nuclear DAPI (blue). **(A)** Mock transfected HeLa cells did not display any endogenous tau staining. **(B)** HeLa cells transfected with full-length 4-repeat (4R) human tau (Fl-Tau) showed the diffuse cytoplasmic distribution of the 4R tau and tau was partially merged with BiP. **(C)** Fl-Tau with human SUMO1 fused to its C-terminus was observed as primarily diffuse cellular staining for tau as well as Fl-Tau but with some perinuclear localization. **(D)** The Fl-Tau SUMO2 fusion protein was distributed within the cytoplasm similar to Fl-Tau. **(E)** Mock transfected HeLa cells probed for BiP, tau and DAPI. **(F)** N-terminally truncated human tau (Tr-Tau) displayed typical cytoplasmic distribution. **(G)** Tr-Tau with SUMO1 fused to its C-terminus showing the accumulation of the fusion protein primarily with a reticular distribution as well as punctate nuclear staining. **(H)** The truncated tau-SUMO2 fusion protein revealed normal cytoplasmic distribution. Scale bar is 20 μm

SUMO1-induced tau misfolding was assessed by immunofluorescence to examine the subcellular localization of the tau-SUMO fusion proteins as compared to the unmodified tau. Mock transfected HeLa cells did not display any endogenous tau staining (Fig. 5A, E). Transient transfection with the unmodified full-length tau (Fl-Tau) indicated a diffuse cytoplasmic distribution and a small amount of overlap with the ER-marker, binding immunoglobulin protein (BiP) or the nucleus as indicated by DAPI staining (Fig. 5B). Some perinuclear staining was observed for the full-length tau-SUMO1 (Fl-Tau-SUMO1) fusion indicating that some proportion of the total protein may undergo misfolding and possibly oligomerization due to the effects of SUMO1 (Fig. 5C). A cytoplasmic localization was seen for the full-length tau-SUMO2 (Fl-Tau-SUMO2) suggesting that it behaves in a similar fashion to the non-SUMOylated tau (Fig. 5D).

**Fig. 6:**
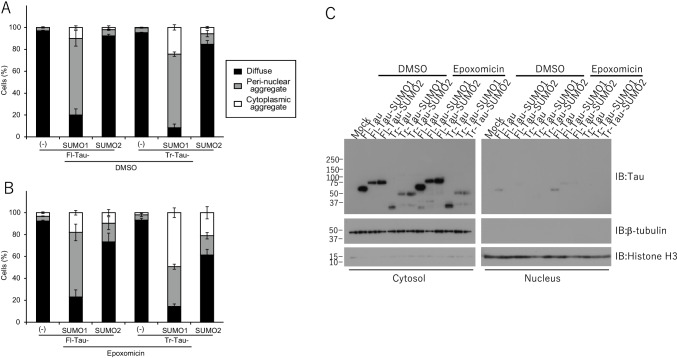
SUMO-tau protein subcellular localization in cells. Morphology and subcellular localization of tau and tau-SUMO fusion proteins were quantified for diffuse, perinuclear and aggregated foci. **(A)** Untransfected control HeLa cells and transfected with full-length 4-repeat (4R) human tau (Fl-Tau). **(B)** HeLa cells and PSP-related N-terminally truncated human tau (Tr-Tau). For quantification, 100 cells/slide and 5 independent slides for each condition were assessed (total 500 cells/condition). (**C**) Fractionation of cytosol and nucleus was performed and Tau, cytosolic marker b-tubulin and nuclear marker Histone H3 were detected by immunoblotting

Transfection of the truncated tau protein (Tr-Tau) indicated a primarily cytoplasmic localization which was comparable to the full-length protein (Fig. 5F). In contrast, the PSP-truncated tau-SUMO1 (Tr-Tau-SUMO1) fusion protein exhibited a partial ER localization that overlapped with BiP staining and primarily with what appeared to be a reticular distribution in the perinuclear space (Fig. 5G). In addition, punctate nuclear staining was observed for the Tr-Tau-SUMO1 fusion suggesting possible trafficking and accumulation in nuclei. To confirm this, we examined the expression of tau in cytosolic and nuclear fractions. As shown in Fig. 6C, tau bands were very faint in nuclear fractions. The truncated tau-SUMO2 (Tr-Tau-SUMO2) resulted in a diffuse cytoplasmic trafficking consistent with a properly folded and trafficked fusion protein (Fig. 5H). Quantification of the number of cells containing diffuse, perinuclear and immunostaining corresponding to aggregated species confirmed the predominant effects were associated with the Tr-Tau-SUMO1 fusion protein (Fig. 6A). These findings indicate that PSP-truncated tau when modified by SUMO1 results in an unstable, misfolded protein that is potentially more prone to form oligomers.

**Fig. 7:**
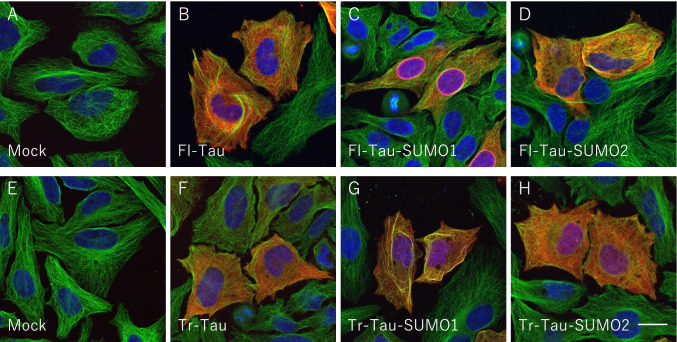
Tau-SUMO co-localization with b-tubulin. Subcellular localization of tau and tau-SUMO fusion proteins in HeLa cells. Cells were stained for b-tubulin (green), tau (red) and nuclear DAPI (blue). **(A)** Mock transfected HeLa cells did not display any tau immunoreactivity. **(B)** HeLa cells transfected with full-length 4-repeat (4R) human tau (Fl- Tau) showed the diffuse cytoplasmic distribution of the unmodified Fl-Tau. **(C)** FL-Tau-SUMO1 exhibited a mixture of cytoplasmic localization and colocalization with b-tubulin in the perinuclear space. **(D)** Fl-Tau-SUMO2 fusion proteins displayed a primarily diffuse cellular staining for tau with no significant overlap with b-tubulin or DAPI. **(E)** Mock transfected HeLa cells were negative for tau. **(F)** PSP-related N-terminally truncated human tau (Tr-Tau) lacking SUMO displayed typical cytoplasmic distribution. **(G)** Tr-tau with SUMO1 fused to its C-terminus (Tr-Tau-SUMO1) displayed significant overlap with b-tubulin. **(H)** Truncated tau-SUMO2 (Tr-Tau-SUMO2) fusion protein showed a predominantly cytoplasmic distribution. Scale bar is 20 μm

The reticular staining observed for the Tr-Tau-SUMO1 protein raised the possibility that it may be co-localizing with cytoskeletal structures within the cells. To investigate this further, cells expressing the full-length and truncated tau proteins were double-labeled for β-tubulin. Immunofluorescence of mock transfected HeLa cells indicated the typical tubulin staining associated with microtubules (Fig. 7A, E). Transfection with Fl-Tau or the Fl-Tau-SUMO1 or Fl-Tau-SUMO2 fusion proteins were found to be diffusely distributed in the cytoplasm with some perinuclear localization of the Fl-Tau-SUMO1 fusion protein (Fig. 7B-D). Similar staining was also observed for the Tr-Tau and Tr-Tau-SUMO2 proteins (Fig. 7F, H). By comparison, the Tr-Tau-SUMO1 protein exhibited a high degree of overlap with tubulin (Fig. 7G). Co-localization with tubulin appeared to be specific in this case as no overlap was observed for either the Fl-Tau or Tr-Tau with intermediate filaments as determined by staining for vimentin (Supplemental Fig. S1). These findings suggest that the PSP-related tau fragment, Tr-Tau, when fused with SUMO1 displayed a high propensity to bind to and co-localize with tubulin. The aggregation and accumulation of the Tr-Tau-SUMO1 are consistent with previous reports where N-terminally cleaved tau was found to increase oligomerization as well as upregulate site-specific phosphorylation [22]. The association with microtubules is also in keeping with studies which have demonstrated a high binding of truncated tau species to microtubules leading to greater stability [21]. The findings of the current investigation indicate that these properties of truncated tau are enhanced by SUMO1 modification.

### Tau SUMOylation and Proteasome-mediated Degradation

The ubiquitin–proteasome system (UPS) plays a key role in the degradation of aggregated proteins and is a significant contributing factor in several neurodegenerative disorders [41]. The UPS is one of the pathways by which tau is degraded and has been linked to Alzheimer’s disease pathogenesis [42]. The potential effects of impaired proteasome degradation were therefore examined using the inhibitor, epoxomicin, in combination with the tau-SUMO fusion proteins.

**Fig. 8:**
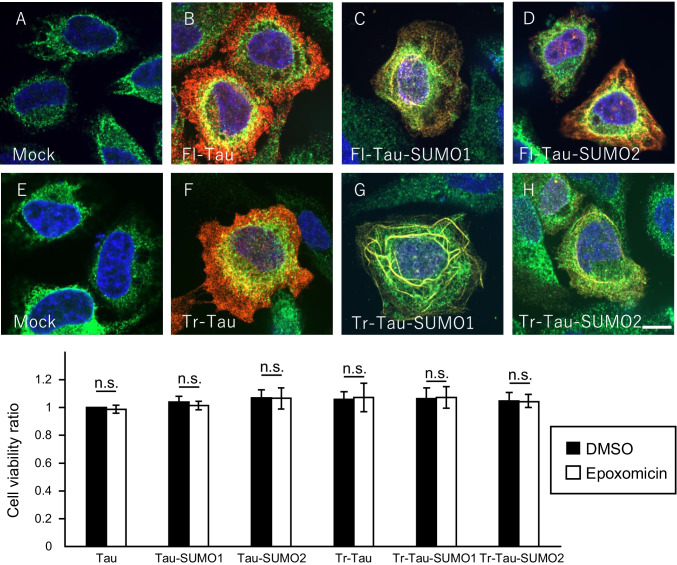
Proteasome inhibition increases Tau-SUMO1 accumulation. Subcellular localization of tau and tau-SUMO fusion proteins in HeLa cells treated with epoxomicin (0.1 μM, 24 h). Cells were stained for the ER-marker BiP (*green*), tau (red) and nuclear DAPI (*blue*). **(A)** Mock transfected HeLa cells stained for the ER-marker BiP (green) and nuclear DAPI (blue) did not display any tau immunoreactivity. **(B)** HeLa cells transfected with full-length 4-repeat (4R) human tau (Fl-Tau) showed the diffuse cytoplasmic distribution of the unmodified Fl-Tau and tau was partially merged with BiP. **(C)** FL-Tau-SUMO1 in the presence of epoxomicin exhibited cytoplasmic distribution with some reticular accumulation. **(D)** Fl-Tau-SUMO2 fusion proteins displayed a primarily diffuse cellular staining for tau. **(E)** Mock transfected HeLa cells were negative for tau. **(F)** PSP-related N-terminally truncated human tau (Tr-Tau) lacking SUMO displayed typical cytoplasmic distribution. **(G)** Tr-tau with SUMO1 fused to its C-terminus (Tr-Tau-SUMO1) accumulated in a reticular pattern suggesting microtubule association. **(H)** Truncated tau-SUMO2 (Tr-Tau-SUMO2) fusion protein showed mainly cytoplasmic distribution and partially reticular formation. (**I**) Cell viability was checked by CellTiter-Glo assay. There was no significant deference between epoxomicin(-) and epoxomicin( +) cells (student’s t-test, p > 0.05). Scale bar is 20 μm

Untransfected HeLa cells treated with epoxomicin exhibited no overt changes in viability and no endogenous tau was observed (Fig. 8A, E). The distribution of Fl-Tau was primarily cytoplasmic but some overlap with the ER-marker BiP was detected (Fig. 8B). In contrast, epoxomicin treatment of cells expressing the Fl-Tau-SUMO1 fusion protein was distributed within the cytoplasm with some reticular accumulation (Fig. 8C). This was not observed for untreated Fl-Tau-SUMO1 expressing cells and suggests that the combination of SUMO1-induced misfolding and reduced proteasome-mediated clearance promotes tau accumulation. This effect was not as evident for the Fl-Tau-SUMO2 fusion protein which had a cytoplasmic distribution similar to the unmodified Fl-Tau (Fig. 8D). However, a small percentage of cells expressing Fl-Tau-SUMO2 exhibited reticular staining suggesting that conjugation of SUMO2 to tau may result in a proportion of aggregated protein. Cumulatively, these findings indicate that SUMO1 has the strongest effect on tau with respect to the promotion of oligomerization and cellular accumulation.

Inhibition of the proteasome was also investigated with the PSP-related Tr-Tau to determine the effects of reduced clearance coupled with SUMOylation. Cells transiently transfected with Tr-Tau and treated with epoxomicin appeared to be very similar to Fl-Tau with primarily cytoplasmic distribution and a modest amount of ER co-localization (Fig. 8F). The Tr-Tau-SUMO1 fusion protein displayed the most pronounced response to epoxomicin treatment with some cytoplasmic staining but the majority of the Tr-Tau-SUMO1 exhibited a reticular distribution consistent with microtubule accumulation (Fig. 8G). The truncated protein appeared to have a much greater propensity to undergo mislocalization. Unlike the Fl-Tau-SUMO2 fusion, the Tr-Tau-SUMO2 also exhibited a tendency toward accumulation but to a lesser extent than the Tr-Tau-SUMO1 (Fig. 8H). As with the untreated cells, quantification of the epoxomicin-treated cells for diffuse, perinuclear and aggregated tau proteins confirmed that the most significant effects were observed for the Tr-Tau-SUMO1 fusion protein (Fig. 6B). To examine the proteasome inhibitor could cause not only perinuclear but also nuclear localization of tau, we observed tau expression in cytosolic and nuclear fractions. As shown in Fig. 6C, the inhibitor did not affect nuclear expression of tau (Fig. 6C). Proteasome inhibition also resulted in extensive co-localization of the Tr-Tau-SUMO1 with microtubules and to a lesser degree for the other tau species (Supplemental Fig. S2). To confirm the effects of cell toxicity of epoxomicin on the localization of tau, we examined cell viability of each tau construct expressing cells with or without epoxomicin. There was no effect of epoxomicin on the cell viability (p > 0.05, Student’s *t* test) (Fig. 8I). This suggests that the truncated tau associated with PSP is more prone to associate with and accumulate on microtubules that is enhanced by SUMO1 modification.

**Fig. 9:**
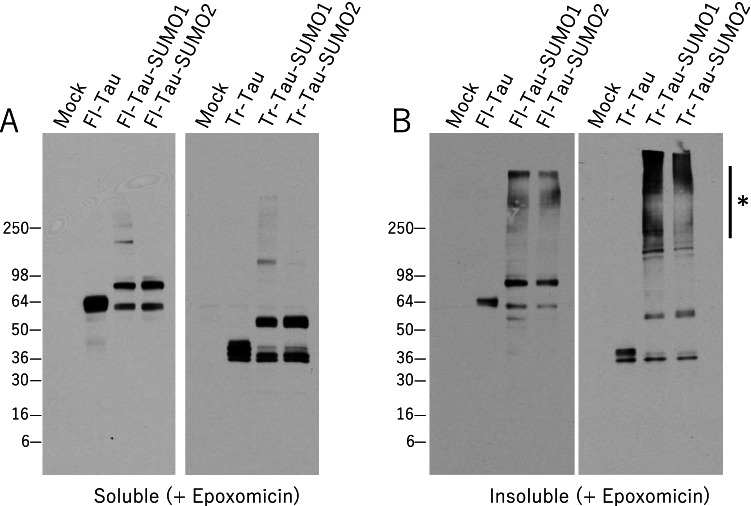
Effects of proteasome inhibition on Tau-SUMO fusion protein aggregation. HEK293 cells transfected with empty vector (Mock), full-length human tau (Fl-Tau), Fl-Tau-SUMO1, Fl-Tau-SUMO2, PSP truncated tau (Tr-Tau), Tr-Tau-SUMO1 or Tr-Tau-SUMO2 fusion proteins with proteasome inhibitor epoxomicin (1 μM, 24 h). **(A)** Extraction with Triton X-100 indicated a significant proportion of soluble tau and tau-SUMO proteins. **(B)** Examination of the insoluble fraction indicated the presence of some Fl-Tau and higher molecular weight aggregates of the Fl-Tau-SUMO1/2 fusion proteins. Significantly greater amounts of the Tr-Tau-SUMO1 were observed in the insoluble fraction and to a lesser extent for the Tr-Tau-SUMO2 fusion protein as indicated by the presence of higher molecular weight insoluble aggregates (*)

Cells expressing each tau construct with proteasome inhibition were also examined by biochemical fractionation to support the immunofluorescence findings. Following the 24 h epoxomicin treatment, a significant amount of the Fl-Tau as well as the Fl-Tau-SUMO1 and Fl-Tau-SUMO2 fusion proteins remained in a soluble fraction (Fig. 9A). Similar levels of soluble fractions were observed for the Tr-Tau and the two fusion proteins, Tr-Tau-SUMO1 and Tr-Tau-SUMO2 (Fig. 9A). Proteasome inhibition did, however, result in the accumulation of Tau-SUMO proteins in insoluble fractions. A modest amount of the Fl-Tau was observed and this appeared as a single, presumably monomeric, species after when solubilized in SDS-PAGE sample buffer (Fig. 9B). Similar monomeric bands were seen for the Fl-Tau-SUMO1 and Fl-Tau-SUMO2 fusion proteins but also higher molecular weight smears indicative of aggregated and oligomeric species that remained insoluble (Fig. 9B). The Fl-Tau-SUMO1 fusion displayed a higher number of aggregates as compared to the corresponding SUMO2 fusion consistent with the immunofluorescence results. However, this aggregation phenomenon was most evident for the truncated tau-SUMO proteins. The Tr-Tau-SUMO1 exhibited the most dramatic degree of aggregation following proteasome inhibition with the majority of the transfected protein appearing as high molecular weight species. Aggregated and insolubleTr-Tau-SUMO2 fusion protein but were less abundant as compared to Tr-Tau-SUMO1. On the other hand, unmodified Tr-Tau did not show higher molecular weight species (Fig. 9B). The increased aggregation of the truncated tau fusion proteins, particularly the SUMO1 fusion, indicates that comparable SUMOylation in PSP may be a significant factor in the accumulation that would be exacerbated by proteasome impairment.

## Discussion

SUMO1-positive inclusions have been reported for multiple system atrophy (MSA), diffuse Lewy body disease (DLB) and PSP where it is found in lysosomal compartments based on cathepsin D co-localization [23, 43]. In this study, we detected SUMO1-positive inclusions in PSP as well as previous study. In addition, we have confirmed the SUMO1 modification of Tr-Tau in PSP brain by immunoprecipitation assay. In the previous reports, punctate structures also contain Hsp90 possibly reflecting attempts by the cells to reverse the protein aggregation process. Comparable protein inclusions could be promoted in vitro using oligodendroglial cultures treated with proteasome inhibitions [23]. This appears to be a common feature for many aggregation-related disorders. In the case of neuronal intranuclear inclusion disease (NIID), the constituent proteins have not been identified but protein deposits display significant SUMO1-positive inclusions [44]. The current study has demonstrated that a truncated tau found in fibrillar aggregates is modified by SUMO1 which may increase insolubility leading to deposition.

This relationship of SUMOylation to neurodegeneration has been the subject of several previous studies on related proteins such as α-synuclein where both increased and decreased solubility have been observed [28, 29, 31]. Reduced protein solubility following SUMOylation has also been reported for the huntingtin (Htt) protein where PIAS1-mediated modification by SUMO2 regulated protein accumulation in vitro similar to that observed following proteasome inhibition [30]. In addition, SUMO2-modified Htt is primarily found in the insoluble fractions from post-mortem tissue from Huntington’s disease cases suggesting that SUMOylation is involved in the pathogenic deposition aggregated proteins. However, in our current study indicated the strong involvement of SUMO1, not SUMO2, in PSP pathology.

Directly relevant to PSP and other tauopathies is an in vitro study which has shown that SUMO1 conjugation to tau at K340 results in decreased solubility and the inhibition of normal protein clearance mechanisms [12]. Mechanistically, SUMO1 was found to compete with ubiquitination and its subsequent degradation by proteasome-mediated pathways. The competing activity of SUMO for ubiquitination sites could result in a reduction in normal tau removal due to loss of proteasome recognition. This is supported by a recent study demonstrating that conjugation of SUMO1 to tau results in a decreased susceptibility to proteasome degradation which may promote neuronal accumulation [12]. As shown in the results, the tau-SUMO1 fusion proteins, particularly the PSP-truncated species, have shown that inhibition of proteasome activity results in significantly increased aggregation. SUMOylation can also enhance tau phosphorylation which was upregulated following exposure of cells to Aβ oligomers. A similar increase in phosphorylated tau was observed in APP transgenic mouse models as determined by immunohistochemistry [45]. These findings are consistent with a pathological effect of SUMO1 modification of tau that could contribute to the formation of neurofibrillary tangles in PSP.

Tau is a substrate for SUMOylation which has been shown both in vitro and a SUMO1 transgenic mouse model [8, 9]. However, it is possible that SUMO-related pathways have an indirect impact on tau that contributes to neurofibrillary tangle formation. It has been demonstrated, for example, that SUMOylation binding to the endosomal sorting complexes required for transport (ESCRT) results in an impairment in the vesicular trafficking and secretion of α-synuclein [46]. This could conceivably contribute to the accumulation of protein inclusions such as Lewy bodies and/or alter prion-like propagation of α-synuclein aggregates. Similar but as yet unexplored consequences of SUMOylation by these types of events may also be occurring with respect to tau. This could, for example, include SUMOylation of phosphatases or kinases that alters tau hyperphosphorylation and it will be of interest to investigate potential SUMO conjugates within these types of pathways. However, the direct SUMO1 modification of tau in the PSP isolates shown in Fig. 3B indicates that it may have more of a direct effect on the physical properties of tau leading to its accumulation.

With respect to the direct effects of SUMO1 conjugation to tau, a potential contributing factor in this process may be the predominance of four repeat (4R) tau in PSP rather than 3R isoforms [47]. Previous mutation studies have revealed that tau is almost exclusively modified by SUMO1 at lysine 340 (K340) which is located within the fourth microtubule-binding repeat [9]. It is possible that SUMO1 modification of 4R tau generates a protein complex that is more prone to misfolding and aggregation. It would therefore be of interest to determine if conjugation of SUMO1 to 3R tau resulted in substantially different responses as compared to the 4R splice version. In addition to the alterations in alternate splicing, tau has been shown to undergo proteolytic processing in these disorders which appears to further complicate the aggregation process. PSP is associated with a truncated tau species in the range of 33–34 kD that results from the removal of the N-terminal ~ 160 residues [37, 38, 48]. This would account for the observed lower molecular weight bands observed in the isolated tau aggregates from PSP of which a large proportion have been shown to be modified by SUMO1 in the present study. This mechanism is supported by the observations of the tau-SUMO1 fusion protein model which displayed a propensity to accumulate intracellularly and associated with microtubules. This effect was most pronounced for the truncated tau-SUMO1, even in the absence of proteasome inhibition, and to a much lesser extent for either the full-length tau-SUMO1 fusion or the truncated tau alone which remained largely unchanged. Thus, the combination of SUMO1 conjugation with the truncation of tau appears to exacerbate aggregation and binding to tubulin.

Tau protein undergoes multiple cleavages and the truncated fragments of tau play a role in disease progression and neurodegeneration. For example, two C-terminal cleavage sites, Glu391 and Asp421, by caspases are well characterized and are found in tangles as well as non-fibrillar aggregates [49]. Proteolytic cleavage of tau is also mediated by calpains, thrombin and additional proteases (for review see, [50]). Truncated tau species are observed in NFT and the density of NFT composed of the truncated tau correlate with clinical severity and Braak staging in AD [51]. Tau cleaved at Ila151 affects its subcellular localization and the 151–391 4R tau is dislocated to nucleus [52]. Proteomic investigations have identified several new N-terminally truncated tau species such as the cleavage at Gln124 [21]. The Gln124 and Met11 truncated proteins also displayed stronger microtubule binding and stabilization. In our study, the preferential association of truncated tau was enhanced by SUMO1 modification as seen with the fusion proteins while SUMO2 had less of an effect in vitro. However, it is not clear whether the SUMOylation occurs before or after cleavage of tau. To confirm if SUMOylation regulates not only tau localization but also tau cleavage, the issue should be elucidated in the future.

The current study has demonstrated that tau deposits in PSP brain exhibit immunoreactivity for SUMO1. Biochemical analysis of isolated fibrillar aggregates from PSP tissue has revealed that these are specifically modified by SUMO1. Analysis of tau-SUMO fusion proteins revealed that the N-terminal tau proteolysis and SUMO1 conjugation results increased aggregation and a strong association with microtubules. When considered in the context of other in vivo and cellular investigations, these findings support the notion that modification of tau by SUMO1 contributes to its aggregation which is further aggravated by the inhibition of proteasome-mediated clearance. The unique combination of SUMO1 conjugation and tau truncation may therefore play a role in PSP pathogenesis.

## Supplementary Information

Below is the link to the electronic supplementary material.

Supplementary file1 (PDF 1.01 MB)

## Data Availability

All data generated or analyzed during this study are included in this published article and its supplementary information files. Data will be made available from the corresponding author on reasonable request.
