# Supplementary material for: SUMO1 Modification of Tau in Progressive Supranuclear Palsy
Source: Mol Neurobiol. 2022 May 14;59(7):4419–35. doi: 10.1007/s12035-022-02734-5 (PMC9167224; doi:10.1007/s12035-022-02734-5)
Supplement: Supplementary file 1 — Supplementary file1 (PDF 1.01 MB) [file 12035_2022_2734_MOESM1_ESM.pdf]

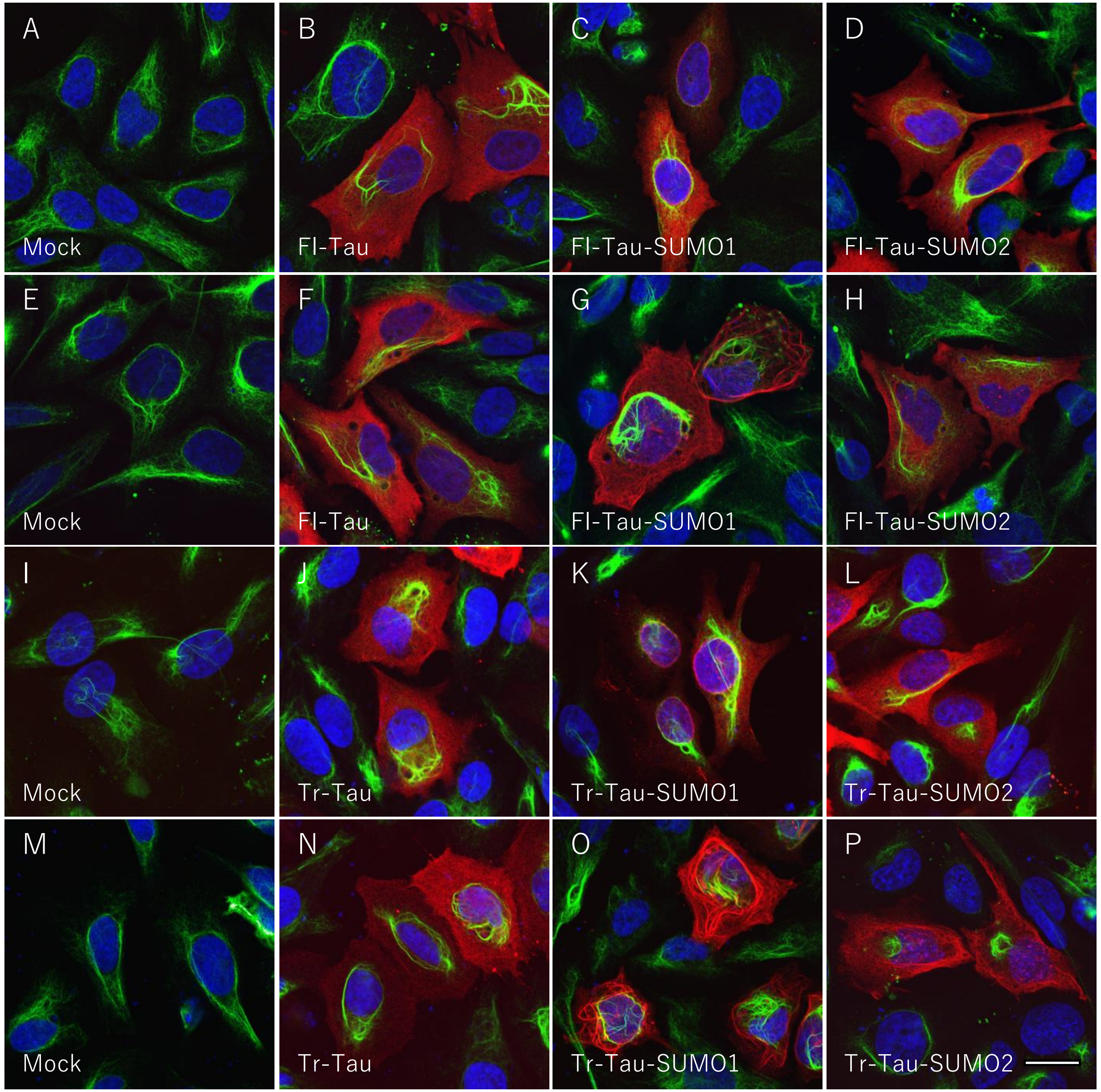

Supplemental Figure S1. Tau-SUMO1 proteins do not co-localize with intermediate filaments. Staining for tau (red), vimentin (green) and DAPI (blue) in HeLa cells that were **(A)** Mock transfected; **(B)** transfected with full-length tau (Fl-Tau); **(C)** Fl-Tau-SUMO1; **(D)** Fl-Tau-SUMO2 proteins without epoxomicin and **(E-H)** the same conditions treated with epoxomicin (0.1 µM, 24 hrs). Cells were **(I)** Mock transfected; **(J)** transfected with truncated tau (Tr-Tau); **(K)** Tr-Tau-SUMO1; **(L)** Tr-Tau-SUMO2 fusion proteins and (M-P) cells under the same conditions in the presence of epoxomicin. No overlap was observed between the diffuse or misfolded tau proteins and the vimentin of intermediate filaments. Scale bar is 20 µm.

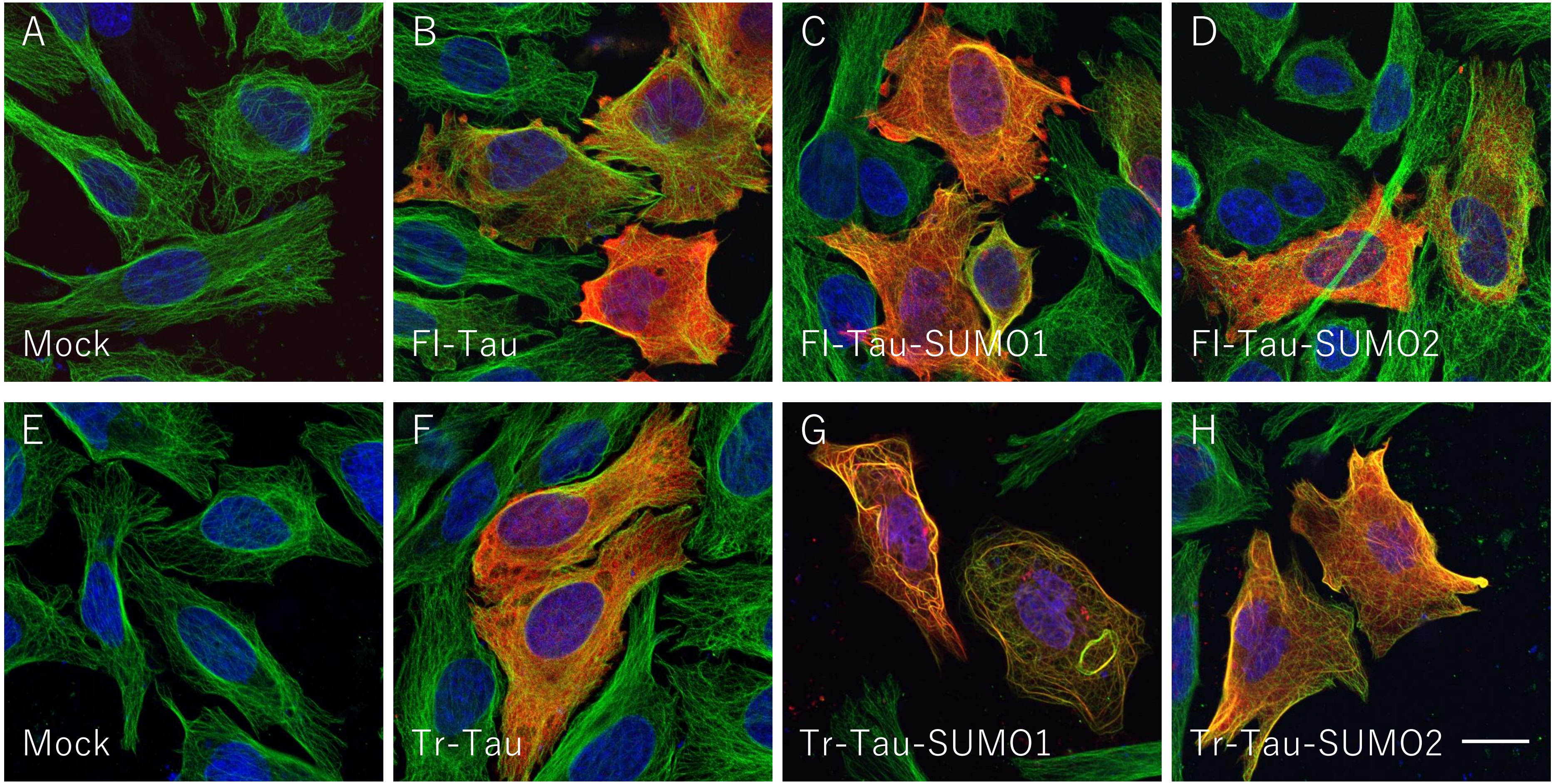

Supplemental Figure S2. Proteasome inhibition and co-localization of SUMO-tau proteins with microtubules. Transfected HeLa cells were treated with epoxomicin (0.1 μM) for a period of 24 hrs. Cells were stained for β-tubulin (green), tau (red) and nuclear DAPI (blue). **(A)** Immunofluorescence of mock transfected HeLa cells indicated typical β-tubulin staining. **(B)** Unmodified Fl-Tau showed the diffuse cytoplasmic distribution. **(C)** FL-Tau-SUMO1 exhibited a mixture of cytoplasmic localization and colocalization with β-tubulin. **(D)** Fl-Tau-SUMO2 fusion proteins displayed a primarily diffuse cellular staining for tau with partial overlap with β-tubulin. Epoxomicin treatments of **(E)** mock transfected cells showed only tubulin staining. Cells expressing the **(F)** PSP-related truncated tau (Tr-Tau) displayed no co-localization with tubulin while the **(G)** Tr-Tau-SUMO1 fusion protein displayed extensive overlap with microtubules. **(H)** The Tr-Tau-SUMO2 protein also co-localized with tubulin but to a lesser extent. Scale bar is 20 μm.
